# Carbon Nitride Materials as Efficient Catalyst Supports for Proton Exchange Membrane Water Electrolyzers

**DOI:** 10.3390/nano8060432

**Published:** 2018-06-13

**Authors:** Ana Belen Jorge, Ishanka Dedigama, Thomas S. Miller, Paul Shearing, Daniel J. L. Brett, Paul F. McMillan

**Affiliations:** 1 Materials Research Institute, School of Engineering and Materials Science, Queen Mary University of London, Mile End Rd, London E1 4NS, UK; 2 Electrochemical Innovation Lab, Roberts Building, Department of Chemical Engineering, University College London, Torrington Place, London WC1E 7JE, UK; ishanka.dedigama.09@ucl.ac.uk (I.D.); t.miller@ucl.ac.uk (T.S.M.); p.shearing@ucl.ac.uk (P.S.); d.brett@ucl.ac.uk (D.J.L.B.); 3 Christopher Ingold Building, Department of Chemistry, University College London, 20 Gordon Street, London WC1H 0AJ, UK; p.f.mcmillan@ucl.ac.uk

**Keywords:** carbon nitride, electrocatalyst, support, water electrolyzer, oxygen evolution reaction, water oxidation

## Abstract

Carbon nitride materials with graphitic to polymeric structures (gCNH) were investigated as catalyst supports for the proton exchange membrane (PEM) water electrolyzers using IrO_2_ nanoparticles as oxygen evolution electrocatalyst. Here, the performance of IrO_2_ nanoparticles formed and deposited in situ onto carbon nitride support for PEM water electrolysis was explored based on previous preliminary studies conducted in related systems. The results revealed that this preparation route catalyzed the decomposition of the carbon nitride to form a material with much lower N content. This resulted in a significant enhancement of the performance of the gCNH-IrO_2_ (or N-doped C-IrO_2_) electrocatalyst that was likely attributed to higher electrical conductivity of the N-doped carbon support.

## 1. Introduction

Among the different ways to produce high-purity hydrogen, proton-exchange water electrolysis currently constitutes the most promising and efficient solution. First developed by General Electric Co. in 1966 for space applications [1], to date, proton-exchange membrane (PEM) water electrolyzers (PEMWEs) have only been used for laboratory-based hydrogen generation and a few large-scale applications. Hydrogen generated via electrolysis can potentially result in zero CO_2_ emissions if the source of electricity is renewable. Moreover, the hydrogen produced is 100% pure and could be used for fuel cell vehicles without further treatment. However, only ~4% of hydrogen is produced from water electrolysis and other lower-cost methods are preferred, such as steam reforming of natural gas [2]. However, as we move towards greater use of renewable energy sources for electrical energy production, the role of electrolysis is set to become increasingly important. Nonetheless, poor anode kinetics, challenging membrane electrode assembly (MEA) preparation and the need for expensive noble metal catalysts and titanium current collectors still hinder the development of this exciting technology and these must be addressed in order to move PEM water electrolyzers forward.

In a water electrolyzer system, the water is split into hydrogen and oxygen. However, this reaction is not thermodynamically favorable and the application of external electrical energy (Δ*H_R_*) is required. The standard potential of a water electrolysis cell is −1.23 V (E^0^_cathode_ − E^0^_anode_) versus SHE (the standard hydrogen electrode). In practice, the process is kinetically controlled, and an overpotential that overcomes losses due to activation energy, charge and mass transfer, surface blockage, and ohmic losses is required for the reaction to take place:

(1) $$H_{2}O_{\left(l\right)}+\mathrm{current}\;\left(\Delta H_{R}\right)\to H_{2}{}_{\left(g\right)}+\frac{1}{2}O_{2}{}_{\left(g\right)},{\;E\;}_{\mathrm{cell}}>-1.23\;V$$

One of the most important obstacles facing PEM electrolysis technology is the use of expensive noble metals such as Pd and Pt at the cathode (for the hydrogen evolution reaction, HER), and Ir and Ru at the anode (for the oxygen evolution reaction, OER). Iridium oxide and ruthenium oxide have been established to be among the most active electrocatalysts for water oxidation, with IrO_2_ showing the best trade-off between performance in acid and alkaline electrolyte solutions [3]. Some of the approaches examined to overcome the high cost of using noble metals include combining IrO_2_ with lower-cost materials such as SnO_2_, Ta_2_O_5_, Nb_2_O_5_, and Co_3_N, among others [4,5,6,7,8,9,10,11,12,13,14,15]. Transition metal perovskites and carbon-based nanomaterials have also been investigated as replacement metal-free catalysts, exhibiting encouraging results [16,17,18,19,20]. However, problems with conductivity, stability, and catalytic activity are still important aspects to address in these alternative systems.

Another way to achieve high activity while reducing the loading of noble metal is the use of advanced catalyst support materials [21,22,23,24,25]. The development of catalyst supports for the anode side (OER) remains a challenge, due to the highly corrosive reaction environment (high potential and acidic electrolyte). Conductive carbons are typically employed in PEM fuel cells, but studies indicate that carbon materials rapidly undergo electrochemical oxidation at the high anodic potentials in the PEM electrolyzers (that operate at >1.5 V versus SHE) [26]. Other support materials that have been explored include metal oxides, carbides, nitrides, and metals [27,28]. The introduction of N into carbon-based supports has been shown to enhance durability, as well as boosting the intrinsic catalytic activity for both oxygen reduction (ORR) and methanol oxidation (MOR) reactions in PEM fuel cells [29,30,31,32,33,34,35,36,37].

Our own work has recently demonstrated that graphitic or polymeric carbon nitride (gCNH) compounds show promising performance characteristics as support materials for Pt catalysts in PEM fuel cells [38,39,40,41,42,43,44].

**Figure 1:**
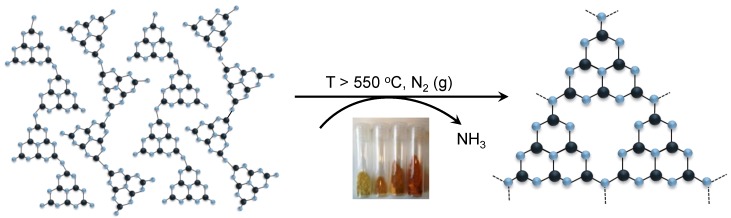
**Left**: The structure of Liebig’s melon that provides a crystalline model for polymeric gCNH structures. The structure contains chains of linked heptazine (tri-*s*-triazine) units; **Right**: When precursors such as melamine, dicyandiamide, or urea are treated above 550 °C in an inert (e.g., N_2_) atmosphere, they undergo condensation reactions with the release of ammonia molecules to form series of amorphous or nanocrystalline polymeric C_x_N_y_H_z_ structures known generally as the graphitic carbon nitride (gCNH) family of materials. A drawing of a laterally polymerized unit formed from “sideways” condensation of melon-like polyheptazine chains is shown. Blue balls represent N atoms; black balls represent C atoms; H atoms are not illustrated, for clarity. The picture under the arrows illustrates the deepening color of different gCNH materials formed as a function of increasing synthesis temperature, from left to right.

Composed of C and N atoms, along with some residual amounts of H, gCNHs are semiconducting materials with a band gap of ~2.3 eV [45,46,47,48,49,50]. When they are prepared by thermolysis of C,N-containing molecules, the structures obtained are thought to be based primarily on heptazine (C_6_N_7_) ring units linked by three-coordinated nitrogen and –NH– bridges to form zigzag chains and partial sheet formation [43] (Figure 1). Preliminary results using IrO_2_ supported on polymeric gCNH in a PEMWE cell revealed an enhancement in the charge-transfer resistance as the current density increases when compared to unsupported IrO_2_. This was attributed to a higher active surface area of the catalyst nanoparticles (NP) on the carbon nitride support [38,42]. In the present study, the catalyst formation reaction and its interaction with the support material are examined in more detail, while developing a better understanding of the electrochemical and PEMWE performance.

## 2. Results and Discussion

### 2.1. Preparation of IrO_2_ and gCNH-IrO_2_ Electrocatalysts

The Adams fusion method is widely used to deposit PtO_2_ from a solution of H_2_PtCl_6_ and NaNO_3_ [51,52,53]. This approach results in the formation of Pt(NO_3_)_2_, which is then oxidized to form PtO_2_ nanoparticles (NP) that can be deposited on a support material [51,53].

**Figure 2:**
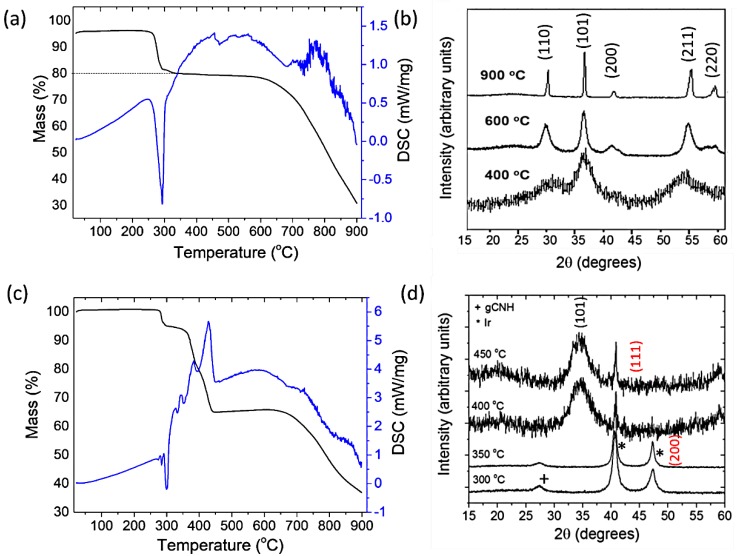
(**a**) TGA/DSC of the reaction between (NH_4_)Ir_2_Cl_6_ and NaNO_3_ conducted in air at a 5 °C min^−1^ heating rate; (**b**) the XRD of the IrO_2_ NPs produced at 400, 600, and 900 °C by the Adams’ fusion method; (**c**) the TGA/DSC of (NH_4_)Ir_2_Cl_6_, NaNO_3_, and gCNH support conducted in air at a 5 °C min^−1^ heating rate; (**d**) XRD of the gCNH-IrO_2_ prepared at 300, 350, 400, and 450 °C. Note: the NaCl formed in the reaction was washed out prior to the XRD acquisition. The weak feature for gCNH occurs at ~27° 2θ. The characteristic strong (111) and (200) reflections for metallic Ir are indexed in red.

In our work, IrO_2_ NPs were produced using a similar approach and the process was monitored using thermogravimetric analysis (TAG) and differential scanning calorimetry (DCS) techniques (Figure 2a). An exothermic peak at ~300 °C was accompanied by a weight loss of ~19%. This process corresponds to the oxidation of (NH_4_)_2_IrCl_6_ to Ir(NO_3_)_4_ accompanied by the formation of NaCl and NH_4_NO_3_:

(2) $${\left({\mathrm{NH}}_{4}\right)}_{2}{\mathrm{IrCl}}_{6}+6{\mathrm{NaNO}}_{3}\to \mathrm{Ir}{\left({\mathrm{NO}}_{3}\right)}_{4}+6\mathrm{NaCl}+2{\mathrm{NH}}_{4}{\mathrm{NO}}_{3}$$

The NH_4_NO_3_ formed then spontaneously decomposes via an exothermic reaction:

(3) $${\mathrm{NH}}_{4}{\mathrm{NO}}_{3}\to N_{2}O+2H_{2}O$$

Above ~640 °C, an abrupt loss of mass is observed associated with the final conversion of Ir(NO_3_)_4_ to IrO_2_.

In order to monitor the degree of crystallinity of IrO_2_ and its crystallite size with the preparation temperature, the synthesis was arrested at different temperatures: 400, 600, and 900 °C. Excess NaNO_3_ along with NaCl that had formed were removed by washing with deionized boiling water, and X-ray diffraction (XRD) patterns for each of the resulting powders were obtained (Figure 2b). By 400 °C, the initial (NH_4_)_2_IrCl_6_ had completely reacted and been oxidized to IrO_2_, and the material exhibited broad diffraction peaks corresponding to an average crystallite size of 4 nm, estimated using the Scherrer formula. IrO_2_ crystallizes in the rutile structure (tetragonal, space group P4 2/mnm, cell parameters a = b ~ 4.49 Å, c ~ 3.14 Å). The broad peaks observed at 32°, 37°, and 55° 2θ in the XRD pattern of the material prepared at 400 °C can be indexed as the (110), (101), and (211) reflections of nanocrystalline IrO_2_, respectively. The formation of IrO_2_ was signaled by an exothermic peak in the DSC diagram, accompanied by a ~19% weight loss in the TGA trace. By 600 °C, the material showing well-defined peaks matching the IrO_2_ rutile crystalline structure had sharpened, indicating a crystallite size that had increased to ~7 nm. At this temperature, additional reflections corresponding to the (200) and (220) reflections of IrO_2_ were also observed at 42° and 59° 2θ, respectively. At 900 °C, the sharp diffraction peaks indicated larger particle sizes on the order of ~30 nm (Figure 2b). No metallic Ir was detected in any of these experiments.

In a previous preliminary study, we had reported the preparation of gCNH-nano-IrO_2_ composites by ball-milling mixtures of the two individual components together in isopropanol [41]. These mechanically produced composites did not exhibit competitive electrocatalytic (EC) performance when tested as anodes in a PEMWE cell, displaying a potential of 2.30 V at 0.42 A cm^−2^, for a gCNH-IrO_2_ (40%) composite compared with 1.85 V obtained for IrO_2_ NPs at the same current density. However, electrochemical impedance measurements did indicate that the gCNH-IrO_2_ composites showed enhanced charge transfer kinetics as the current density was increased, thus, suggesting ways to improve the EC performance [41]. Our results indicated that by developing a method to obtain a better distribution of the IrO_2_ nanoparticles onto the gCNH substrate, a high-performance EC anode behavior could be obtained.

In order to achieve this, we initiated the present study to carry out the formation and deposition reactions of the IrO_2_ nanoparticles onto gCNH support material that was already present in the reaction mixture. For these syntheses, the carbon nitride powder was prepared by thermolysis of dicyandiamide/melamine (1/1) precursors at 550 °C for 15 h under N_2_ (g) and then dispersed in isopropanol before adding (NH_4_)_2_IrCl_4_ and NaNO_3_ to the mixture. The isopropanol was evaporated and the resulting mixture was heated in stages while studying the course of the reaction by TGA/DSC (Figure 2c), with an examination of the products at each stage by XRD, and scanning (SEM) and transmission electron microscopy (TEM). However, an entirely different sequence of events leading to the final formation of IrO_2_ NPs deposited on the catalyst support was observed. At room temperature, the XRD pattern was dominated by reflections from the crystalline starting materials along with a weak broad feature at ~27° 2θ for the gCNH phase (Figure 2d). After heating to 300 °C, the peaks due to (111) and (200) reflections of metallic Ir were observed in the XRD pattern at 41° and 47° 2θ, respectively. Between ~350 and 400 °C, a significant weight loss occurred, while at the same time, the Ir metal became partly oxidized to nanocrystalline IrO_2_, indicated by the broad (101) reflection near 35° 2θ. The weak carbon nitride feature was no longer visible in the pattern. At 400 and 450 °C, the main phase present was IrO_2_ with small nanoparticle size, while XRD reflections from Ir metal remained visible in the pattern recorded at the highest temperature. The large weight loss recorded while the IrO_2_ is being formed can be attributed to the decomposition of the gCNH support material, with the removal of a large proportion of its N component. This is an interesting result because gCNH materials prepared from the thermolysis of precursors are normally found to be stable against decomposition in the air at temperatures up to ~650 °C [46]. Our result shows that the presence and formation of Ir/IrO_2_ NPs in the system can catalyze the decomposition of the carbon nitride support, resulting in a material that corresponds more closely to an electrically conducting N-doped graphite rather than the expected gCNH semiconductor. That would be a desirable outcome for improving the electrochemical performance.

**Figure 3:**
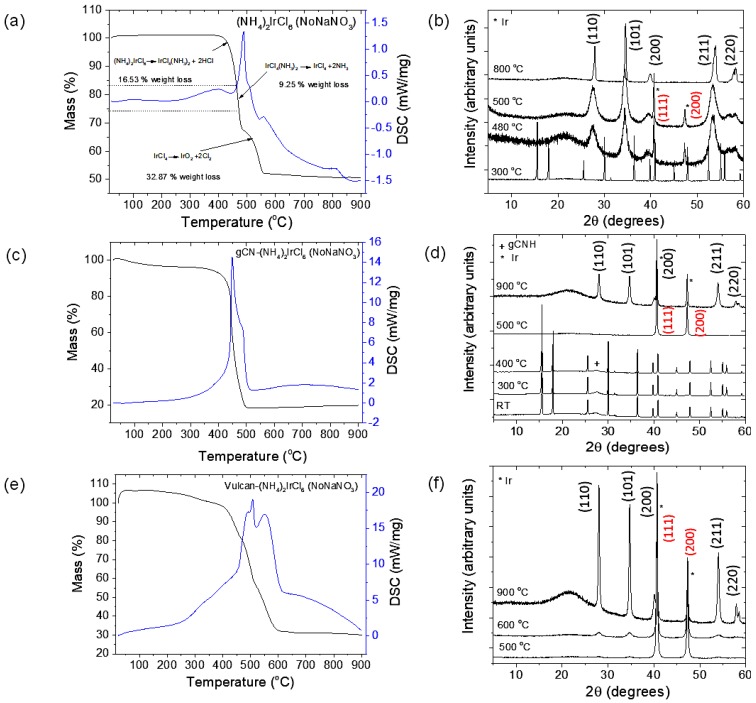
**Left**: TGA/DSC traces of (**a**) (NH_4_)Ir_2_Cl_6_, (**c**) gCNH-(NH_4_)_2_IrCl_6_, (**e**) Vulcan-(NH_4_)_2_IrCl_6_ conducted in air at 5 °C min^−1^ heating rate. **Right:** the XRD of products of TGA analysis at different temperatures for (**b**) (NH_4_)_2_IrCl_6_, (**d**) gCNH-(NH_4_)_2_IrCl_6_ and (**f**) Vulcan-(NH_4_)_2_IrCl_6_. Peaks due to metallic Ir are indexed in red.

In order to better understand the processes associated with the formation of the IrO_2_ nanoparticles, we conducted a TGA/DSC study of the synthesis experiment in the absence of gCNH under the same conditions as those shown in Figure 2a, but without the addition of NaNO_3_ to the mixture (Figure 3a). The NaNO_3_ had been included previously as it was described as an essential reactant in the procedure to prepare nano-PtO_2_ from H_2_PtCl_6_ [54]. In our “No-NaNO_3_” reaction, the formation of IrO_2_ from the salt (NH_4_)_2_IrCl_6_ took place as before, although a different set of processes was observed to occur. In this case, the (NH_4_)_2_IrCl_6_ first undergoes decomposition to IrCl_4_(NH_3_)_2_ with the release of HCl at around 400 °C, followed by the formation of IrCl_4_ and 2NH_3_ at ~450 °C, with the final oxidation to IrO_2_ occurring at ~480–500 °C (Figure 3a). No endothermic peak was observed in the DSC trace, but an exothermic peak appears at ~500 °C. The process was followed by XRD measurements on samples heated to different temperatures: 300, 480, 500, and 800 °C (Figure 3b). By 480 °C, IrO_2_ is already the dominant product, although some Ir metal was also detected in the XRD pattern. However, this component had disappeared by 800 °C and the XRD diagram matched that of pure IrO_2_ (Figure 3b).

A further series of TGA/DSC/XRD experiments were performed to compare the effects of adding gCNH or commercial carbon (Vulcan, Texas, TX, USA) catalyst supports dispersed in isopropanol, again without adding NaNO_3_ to the reaction mixture. In the case of the gCNH-(NH_4_)_2_IrCl_6_ combination, we observed a mass loss of ~80% with onset at 450 °C (Figure 3c), that is, 50% greater than in the absence of the catalyst support medium. We also obtained XRD patterns for the reaction products at different temperatures: 300, 400, 500, and 900 °C (Figure 3d). The gCNH signal had disappeared by 500 °C, at the temperature by which the Ir metal appears in the XRD pattern before there is any indication of IrO_2_ formation. At 900 °C, the appearance of IrO_2_ could be detected by XRD, while some Ir metal also remained present. This behavior is completely different from the sample studied without the presence of gCNH, indicating that the carbon nitride material is an essential participant in the redox reactions. For the Vulcan-(NH_4_)_2_IrCl_6_ system, the TGA/DSC also showed a higher weight loss than in the case of the (NH_4_)_2_IrCl_6_ alone. However, no abrupt loss of weight was observed at 500 °C, but instead, a gradual decrease in mass with increasing temperature was observed (Figure 3e). XRD analysis showed that the Ir metal was formed at 500 °C, but IrO_2_ was also detected, and this became the main product at 900 °C (Figure 3f).

**Figure 4:**
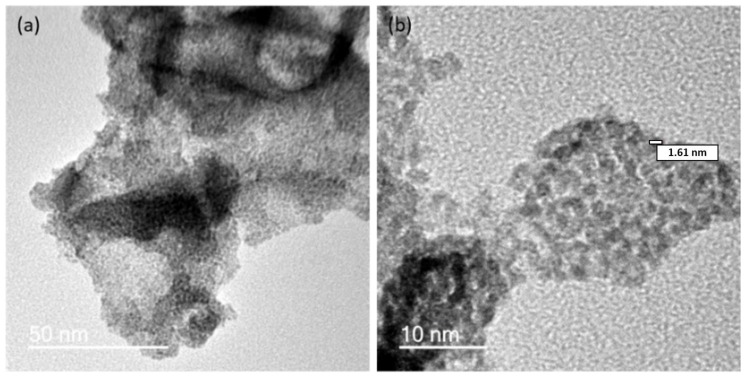
HRTEM images of (**a**,**b**) gCNH-IrO_2_ obtained through Adams’ fusion method at 450 °C.

We obtained HRTEM images to compare the electrocatalyst composites produced by ball-milling a mixture of IrO_2_ and gCNH [40], with those prepared by forming the IrO_2_ NPs in situ in the presence of the gCNH support material (Figure 4). In the case of the samples achieved by mechanical ball-milling, the NPs were not well dispersed, resulting in agglomerates formed on the gCNH. In the case of IrO_2_ prepared and deposited by in situ reaction, the catalyst NPs were well distributed throughout the gCNH support, with a particle size measured to be around 2 nm. The amount of IrO_2_ deposited on the support was noticeably higher in the case of the material prepared using the in situ reaction/deposition method.

**Figure 5:**
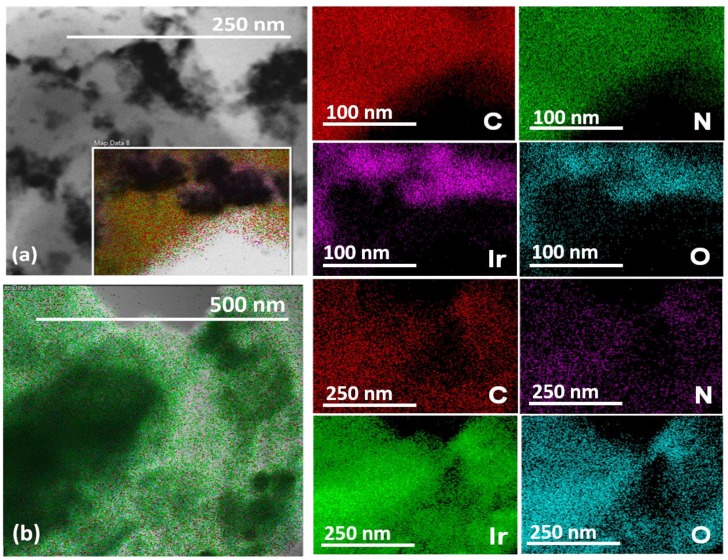
TEM images and corresponding energy-dispersive X-ray spectroscopy (EDX) mapping for (**a**) gCNH-IrO_2_ (40%) prepared by the ball-milling of pre-prepared IrO_2_ and gCNH materials; (**b**) gCNH-IrO_2_ (20%) prepared by in situ deposition of IrO_2_ on gCNH support following an adaptation of Adams’ fusion method at 450 °C.

Energy dispersive X-ray analyses and maps obtained during the TEM studies were used to establish the elemental distribution and composition of the ball-milled versus in situ deposited gCNH-IrO_2_ samples (Figure 5). For the gCNH-IrO_2_ (40%) ball-milled sample the C, N, Ir, and O elemental contents determined by bulk analysis closely matched those of the starting materials, as expected [41]. However, in the case where the IrO_2_ was prepared and deposited in the presence of the gCNH support material, the results are quite different (Figure 4b). First, the N content in the sample close to the IrO_2_ particles was substantially depleted to only a few (e.g., ~3 wt %) compared with a theoretical N composition of 52.7 wt % for the gCNH phase (Figure 5b). This correlates with the results obtained by TGA/DSC and XRD, which suggested a substantial loss of the N-component from the gCNH support. Such an extensive loss of N was confirmed by bulk elemental analysis, where a C/N ratio of ~0.5 was recorded compared with the initial ~1.5 ratio for the starting gCNH compound and observed for the ball-milled materials.

**Figure 6:**
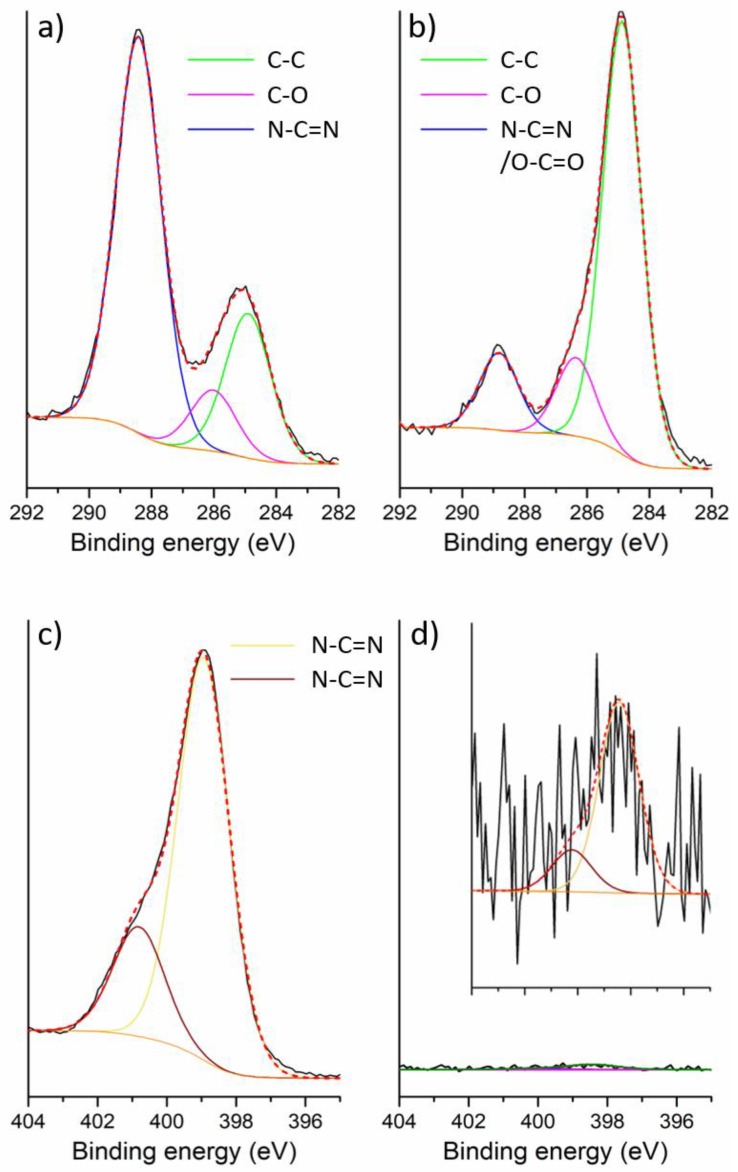
XPS spectra: C 1s (**a**) and N 1s (**b**) spectra of gCNH-IrO_2_ (20 wt %) prepared by ball-milling; C 1s (**c**) and N 1s (**d**) spectra of gCNH-IrO_2_ (450 °C); the inset shows an expansion of the N 1s peak.

X-ray photoelectron spectroscopy (XPS) analyses support this finding (Figure 6). The gCNH-IrO_2_ sample prepared by carrying out the catalytic NP synthesis in the presence of the carbon nitride support at 450 °C clearly shows a dramatically reduced N content at least in the surface region with a N at % of 1.98, compared with that for the ball-milled sample, that retains its original value of 40.26 at % (Figure 6c,d).

The C 1s spectra (Figure 6a,b) for both samples contain the same number of peaks, which can be fit with Gaussian-Lorentzian (GL) contributions at equivalent binding energies. The peak near 288 eV in the ball milled sample is typically assigned to sp^2^ bonded C atoms associated with triazine or heptazine ring units in the carbon nitride (N-C=N) [44]. The other two peaks at the C-C peak at ~284 eV and ~286 eV can be attributed to C-C and C-O bonding environments, either associated with adventitious carbon species or due to the C-C bonding intrinsic to the structure [44]. The magnitude of these peaks is dramatically different between the two samples. In the ball-milled material, the N-C=N carbon peak is dominant and contributes 65 at %, with the C-C peak contributing 24 at %. In the Adams’ fusion material made at 450 °C the carbon peak at 288 eV only accounts for 13 at %, significantly less than the same peak in the ball milled material. The C-C bonded sites represent 73 at % of the sample. In both cases, the C-O peak contributes the remaining ~10 at %.

The N 1s spectra show a similar trend, both exhibiting a dominant feature near 398.8 eV that is assigned to C-N=C units within either triazine or heptazine rings and a smaller contribution at 400.8 eV appearing as a shoulder, indicative of the C-N-H uncondensed amino (-NH_2_) groups, or N atoms bridging between three heptazine rings (N-C3 units) [44]. However, the magnitude of the peaks for the material made by Adams’ fusion method at 450 °C is greatly reduced.

The ratio of the N peak at ~399 eV and the C peak at ~288 eV should be close to 1:1 if both are attributed to C-N=C units, as is the case for the ball milled material. However this ratio changes to 1:13 for the material prepared by the Adams’ fusion method at 450 °C, showing that the C peak cannot be attributed to triazine/heptazine units. This material shows evidence for an increased O concentration so that the major contribution to the peak at ~288 eV can be attributed to carboxy-groups.

### 2.2. Electrochemical Performance of gCNH-IrO_2_ Composites

**Figure 7:**
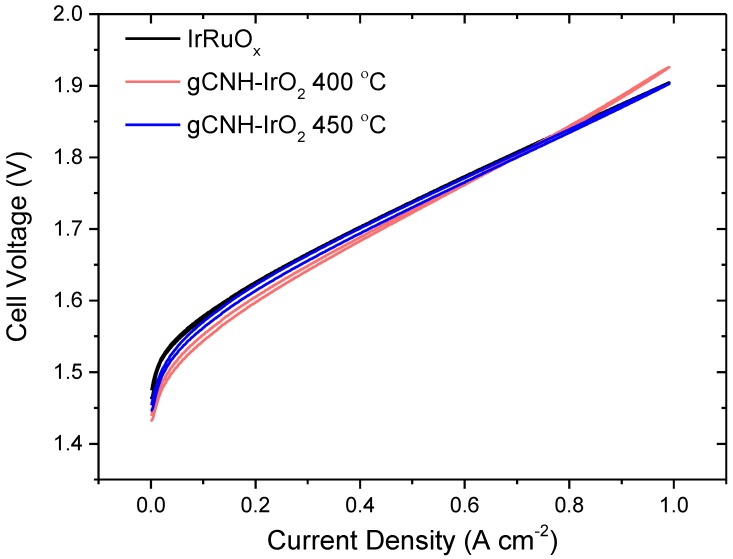
Polarization measurements of PEMWEs with MEAs containing anodes made from (**a**) IrO_2_, (**b**) gCNH-IrO_2_ prepared at 400 °C, and (**c**) gCNH-IrO_2_ prepared at 450 °C. Measurements conducted at 80 °C and atmospheric pressure.

Electrochemical tests of the anode composites were carried out in a PEM water electrolyzer cell. Previous studies have shown that the gCNH-IrO_2_ (40%) prepared by ball milling (1.6 mg cm^−2^) exhibited an onset potential of 1.53 V and potential of 2.30 V at 0.42 A cm^−2^ [41]. This material had poor overall performance, so no data were collected at 1 A cm^−2^ due to the high overpotential already reached at lower current densities. Figure 7 shows the PEM electrolyzer cell polarization measurements for three different MEAs prepared and tested in this study, namely, a commercial MEA with an IrRuO_x_ anode that was used as a benchmark for the study, an MEA with a gCNH-IrO_2_ anode prepared at 400 °C, and an MEA with gCNH-IrO_2_ anode prepared at 450 °C. In all cases, Pt/C was used as the cathode material for the hydrogen evolution reaction.

As shown in the three polarization curves in Figure 7, the MEA produced with the gCNH-IrO_2_ catalyst prepared at 400 °C MEA exhibited excellent performance at low current densities (<0.7 A cm^−2^), with a cell potential of 1.6 V at 0.2 A cm^−2^. As the current density was increased, the cell potential of the gCNH-IrO_2_ at 400 °C MEA also increased, giving a final cell potential of 1.93 V at 1 A cm^−2^, the highest current density tested in these experiments. This value was slightly higher than that obtained for the commercial MEA (1.90 V). In the case of the gCNH-IrO_2_ (450 °C) MEA, although the cell potential is slightly higher than the cell with the gCNH-IrO_2_ (400 °C) MEA, it was lower than the commercial MEA and it showed better performance at higher current densities, achieving cell potential values similar to the commercial IrRuO_x_ MEA.

**Figure 8:**
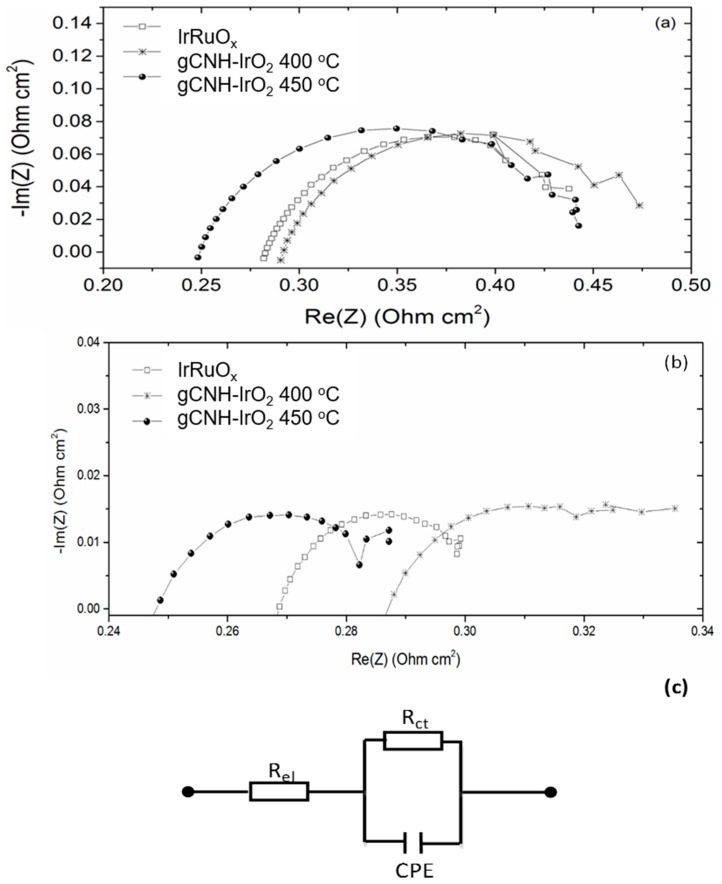
EIS Nyquist plots conducted in a PEM water electrolyzer at 80 °C, using Pt black as the cathode and gCNH-IrO_2_ prepared at 400 and 450 °C as anode at (**a**) 0.1 A cm^−2^ and (**b**) 1 A cm^−2^. Commercial IrRuO_x_ anode results are included for comparison. (**c**) Equivalent circuit used for data fitting. *R_ct_* is the charge transfer resistance described in the text.

The electrochemical performance of the three MEAs was further interpreted by analyzing the electrochemical impedance spectroscopy (EIS) data (Figure 8). It can be seen that the gCNH-IrO_2_ 400 °C MEA exhibits the highest cell resistance (*R_el_*) (0.285 Ω cm^2^) of the three catalysts at a current density of 0.1 A cm^−2^ (baseline catalyst; 0.277 Ω cm^2^ and IrO_2_(20%)-gCNH at 450 °C; 0.243 Ω cm^2^), which explains the higher slope of the polarization curve that results in a higher cell potential at 1 A cm^2^.

The gCNH-IrO_2_ 400 °C MEA exhibited the highest resistance at both current densities considered. This would explain its poorer performance compared to the other two MEAs. The fitted parameters showed that at 0.1 A cm^−2^, the *R_ct_* (charge transfer resistance) of the three MEAs increased from 0.200 Ω cm^2^ (gCNH-IrO_2_ 400 °C) to 0.210 Ω cm^2^ (gCNH-IrO_2_ 450 °C) and 0.216 Ω cm^2^ (IrRuO_x_) (Table 1), which explains the relatively good performance of gCNH-IrO_2_ at 400 °C at a low current density. The EIS spectra recorded at 1 A cm^−2^ exhibited features corresponding to mass transport limitations at low frequencies that can be attributed to the formation of oxygen and hydrogen bubbles in the electrolyzer cell.

## 3. Materials and Methods

### 3.1. Synthesis of gCNH

Polymeric carbon nitride was prepared by thermolysis and condensation reactions of a 1:1 molar ratio mixture of dicyandiamide (C_2_N_4_H_4_, Sigma Aldrich, Dorset, UK) and melamine (C_3_N_6_H_9_, Sigma Aldrich, Dorset, UK) at 550 °C. The finely ground starting mixture was loaded in an alumina boat into a quartz tube in a tube furnace under a flow of nitrogen. The furnace temperature was raised at 5 °C min^−1^ and held for 15 h. The furnace was allowed to cool to room temperature before the product was removed. Further details of the synthesis and characterization of this material are given elsewhere [45].

### 3.2. Synthesis of IrO_2_ Nanoparticles

IrO_2_ was synthesised using an adaptation of Adams’ fusion method that is widely applied to the synthesis of noble metal oxide nanoparticles [51,54,55]. The original method involves fusion of a metal chloride precursor with NaNO_3_ in air at an elevated temperature, resulting in the formation of the metal oxide and NaCl. Following this, a predetermined amount of (NH_4_)_2_IrCl_6_ (Sigma Aldrich, Dorset, UK) was dissolved in isopropanol (Sigma Aldrich, Dorset, UK) to achieve a final metal concentration of 4 × 10^−2^ M, and was magnetically stirred for 2 h. An excess of finely ground NaNO_3_ (Sigma Aldrich, Dorset, UK) was added to the solution, which was further stirred for 1 h. The mixture was thermally treated at 500 °C for 1 h in air. The obtained black powder was washed several times with boiling deionized water to remove the unreacted NaNO_3_. The IrO_2_ powder was dried at 80 °C overnight.

### 3.3. Synthesis of gCNH-IrO_2_

The composite gCNH-IrO_2_ was also prepared following Adams’ fusion method, but with the gCNH material dispersed in isopropanol before the (NH_4_)_2_IrCl_4_ was added. The amount of Ir salt added was calculated to result in 20 wt % IrO_2_ in the final composite. As previously, an excess of finely ground NaNO_3_ was added to the solution, which was further stirred for 1 h. In order to study the effect of the synthesis temperature on the final composite, the mixture was thermally treated at 400 °C and 450 °C for 1 h in air, and the two corresponding materials were labelled as gCNH-IrO_2_ 400 °C and gCNH-IrO_2_ 450 °C. The obtained black powders were then washed several times with boiling deionized water to remove the unreacted NaNO_3_, and dried at 80 °C overnight. The results were compared with those for a commercial IrRuO_x_ electrocatalyst.

### 3.4. Membrane Electrode Assembly (MEA) Preparation

Nafion 115 (thickness ~127 µm, DuPont UK Ltd., Bristol, UK) was used as the PEM for the MEA preparation. Following a standard procedure, the Nafion membrane was pre-treated in hydrogen peroxide (5 wt %) at 80 °C for 1 h to remove organic impurities. After being flushed with deionized water, it was transferred into a 0.5 M sulfuric acid solution and boiled at 80 °C for an additional 1 h to protonate the membrane. Finally, the membrane was washed with deionized water. The catalyst inks were prepared by dispersing the catalyst powder into a mixture of water and Nafion solution 10% (Sigma Aldrich) and ultrasonically dispersed for 2 h before being used. All the MEAs used in this study were prepared by spraying the catalyst ink onto the Nafion membrane using an air-driven spray gun. Catalyst loading for the cathode was 4 mg cm^−2^ of Pt. The active area of the prepared MEAs was 7.07 cm^2^.

### 3.5. Structural and Compositional Characterization

C, N, and H analyses were performed using a Carlo-Erba EA1108 system (Thermo scientific, Waltham, MA, USA). Thermogravimetric analysis (TGA) and differential scanning calorimetry (DSC) analyses were performed on a Netzsch DSC/TGA instrument (Selb, Germany). Transmission electron microscopy (TEM) images were taken using a JEOL JEM2010 instrument (Akishima, Tokyo, Japan) operating at 200 kV; the samples were prepared by dispersing the particles in methanol and evaporating the suspension drops on holey carbon grids (Agar Scientific, Essex, UK).

### 3.6. Electrochemical Performance Evaluation

A PEM water electrolyzer cell supplied by ITM-Power was used to investigate the electrochemical performance of the prepared MEAs at an operating temperature of 80 °C. Preheated deionized water (18.3 MΩ cm) circulated by two peristaltic pumps at a flow rate of 150 mL min^−1^ was supplied to both anode and cathode. Polarization curves were recorded galvanostatically between 0 and 1 A cm^−2^ at a 10^−3^ A s^−1^ scan rate. Electrochemical impedance spectroscopy (EIS) measurements were carried out potentiostatically at 0.1 and 1 A cm^−2^ with an amplitude of 5 mV in the frequency range of 10 MHz to 10 kHz. All electrochemical measurements were performed using an Autolab PGSTAT 30 Potentiostat/Galvanostat (Metrohm UK Ltd., Cheshire, UK) equipped with a frequency response analyzer (FRA).

## 4. Conclusions

gCNH materials were investigated as catalyst supports for PEM water electrolyzers, specifically as support for IrO_2_, catalyzing the water oxidation reaction taking place at the anode. IrO_2_ nanoparticles were successfully synthesized and deposited onto support materials. However, it was discovered that the process of forming these IrO_2_ nanoparticles in situ onto the gCNH support acted to catalyze its decomposition leading to the substantial loss of the N-H component, as corroborated by TGA/DSC, elemental analysis, and XPS and EDS measurements. Together, the results indicate that the carbon nitride has been converted into a highly N-deficient carbon nitride or heavily N-doped graphitic carbon material that should be further investigated in future work. The loss in N from the support resulted in a significant enhancement of the performance of the gCNH-IrO_2_ (or N-doped C-IrO_2_) electrocatalyst that we can attribute to a higher electrical conductivity of the support.

## Figures and Tables

**Table 1:** EIS fitted parameters of the data shown in Figure 8.

	IrRuO_x_		gCNH-IrO_2_-400 °C		gCNH-IrO_2_-450 °C	
	0.1 A cm^−2^	0.7 A cm^−2^	0.1 A cm^−2^	1 A cm^−2^	0.1 A cm^−2^	1 A cm^−2^
*R_el_* (Ω cm^2^)	0.277	0.263	0.285	0.273	0.243	0.235
*R_ct_* (Ω cm^2^)	0.216	0.048	0.200	0.070	0.210	0.058
*CPE* (Ω^−1^s^n^)	0.238	0.518	0.038	0.209	0.033	0.054
*n*	0.72	0.65	0.79	0.55	0.79	0.66

## References

[1] Russell J.H., Nuttall L.J., Fickett A.P. (1973). Hydrogen generation by solid polymer electrolyte water electrolysis. Am. Chem. Soc. Div. Fuel Chem. Prepr..

[2] Haryanto A., Fernando S., Murali N., Adhikari S. (2005). Current status of hydrogen production techniques by steam reforming of ethanol: A review. Energy Fuels.

[3] Fabbri E., Habereder A., Waltar K., Kötz R., Schmidt T.J. (2014). Developments and perspectives of oxide-based catalysts for the oxygen evolution reaction. Catal. Sci. Technol..

[4] De Pauli C.P., Trasatti S. (1995). Electrochemical surface characterization of IrO_2_+SnO_2_ mixed oxide electrocatalysts. J. Electroanal. Chem..

[5] Morimitsu M., Otogawa R., Matsunaga M. (2000). Effects of cathodizing on the morphology and composition of IrO_2_-Ta_2_O_5_/Ti anodes. Electrochim. Acta.

[6] Terezo A.J., Bisquert J., Pereira E.C., Garcia-Belmonte G. (2001). Separation of transport, charge storage and reaction processes of porous electrocatalytic IrO_2_ and IrO_2_/Nb_2_O_5_ electrodes. J. Electroanal. Chem..

[7] Chanda D., Hnát J., Bystron T., Paidar M., Bouzek K. (2017). Optimization of synthesis of the nickel-cobalt oxide based anode electrocatalyst and of the related membrane-electrode assembly for alkaline water electrolysis. J. Power Sources.

[8] Marshall A.T., Sunde S., Tsypkin M., Tunold R. (2007). Performance of a PEM water electrolysis cell using IrxRuyTazO_2_ electrocatalysts for the oxygen evolution electrode. Int. J. Hydrogen Energy.

[9] Audichon T., Mayousse E., Napporn T.W., Morais C., Comminges C., Kokoh K.B. (2014). Elaboration and characterization of ruthenium nano-oxides for the oxygen evolution reaction in a Proton Exchange Membrane Water Electrolyzer supplied by a solar profile. Electrochim. Acta.

[10] Fuentes R.E., Rau S., Smolinka T., Weidner J.W. (2010). Bimetallic electrocatalysts supported on TiO_2_ for PEM water electrolyzer. ECS Trans..

[11] Reier T., Pawolek Z., Cherevko S., Bruns M., Jones T., Teschner D., Selve S., Bergmann A., Nong H.N., Schlögl R. (2015). Molecular insight in structure and activity of highly efficient, low-Ir Ir–Ni oxide catalysts for electrochemical water splitting (OER). J. Am. Chem. Soc..

[12] Tunold R., Marshall A., Rasten E., Tsypkin M., Owe L., Sunde S. (2010). Materials for Electrocatalysis of Oxygen Evolution in PEM Water Electrolysis. Ph.D. Thesis.

[13] Chen D., Chen C., Baiyee Z.M., Shao Z., Ciucci F. (2015). Nonstoichiometric oxides as low-cost and highly-efficient oxygen reduction/evolution catalysts for low-temperature electrochemical devices. Chem. Rev..

[14] Zhang Y., Ouyang B., Xu J., Jia G., Chen S., Rawat R.S., Fan H.J. (2016). Rapid Synthesis of Cobalt Nitride Nanowires: Highly Efficient and Low-Cost Catalysts for Oxygen Evolution. Angew. Chem. Int. Ed..

[15] McAteer D., Godwin I.J., Ling Z., Harvey A., He L., Boland C.S., Vega-Mayoral V., Szydłowska B., Rovetta A.A., Backes C. (2018). Liquid Exfoliated Co(OH)_2_ Nanosheets as Low-Cost, Yet High-Performance, Catalysts for the Oxygen Evolution Reaction. Adv. Energy Mater..

[16] Anantharaj S., Ede S.R., Sakthikumar K., Karthick K., Mishra S., Kundu S. (2016). Recent trends and perspectives in electrochemical water splitting with an emphasis on sulfide, selenide, and phosphide catalysts of Fe, Co, and Ni: A review. ACS Catal..

[17] Han L., Dong S., Wang E. (2016). Transition-Metal (Co, Ni, and Fe)-Based Electrocatalysts for the Water Oxidation Reaction. Adv. Mater..

[18] Wang J., Cui W., Liu Q., Xing Z., Asiri A.M., Sun X. (2016). Recent Progress in Cobalt-Based Heterogeneous Catalysts for Electrochemical Water Splitting. Adv. Mater..

[19] Galán-Mascarós J.R. (2015). Water oxidation at electrodes modified with earth-abundant transition-metal catalysts. ChemElectroChem.

[20] Su D.S., Perathoner S., Centi G. (2013). Nanocarbons for the development of advanced catalysts. Chem. Rev..

[21] Xu J., Liu G., Li J., Wang X. (2012). The electrocatalytic properties of an IrO_2_/SnO_2_ catalyst using SnO_2_ as a support and an assisting reagent for the oxygen evolution reaction. Electrochim. Acta.

[22] Mazúr P., Polonský J., Paidar M., Bouzek K. (2012). Non-conductive TiO_2_ as the anode catalyst support for PEM water electrolysis. Int. J. Hydrogen Energy.

[23] Park S., Shao Y., Liu J., Wang Y. (2012). Oxygen electrocatalysts for water electrolyzers and reversible fuel cells: Status and perspective. Energy Environ. Sci..

[24] Xu J., Aili D., Li Q., Christensen E., Jensen J.O., Zhang W., Hansen M.K., Liu G., Wang X., Bjerrum N.J. (2014). Oxygen evolution catalysts on supports with a 3-D ordered array structure and intrinsic proton conductivity for proton exchange membrane steam electrolysis. Energy Environ. Sci..

[25] Marshall A.T., Haverkamp R.G. (2010). Electrocatalytic activity of IrO_2_–RuO_2_ supported on Sb-doped SnO_2_ nanoparticles. Electrochim. Acta.

[26] Linse N., Gubler L., Scherer G.G., Wokaun A. (2011). The effect of platinum on carbon corrosion behavior in polymer electrolyte fuel cells. Electrochim. Acta.

[27] Antolini A., Gonzalez E.R. (2009). Ceramic materials as supports for low-temperature fuel cell catalysts. Solid State Ion..

[28] Rabis A., Rodriguez P., Schmidt T.J. (2012). Electrocatalysis for polymer electrolyte fuel cells: Recent achievements and future challenges. ACS Catal..

[29] Chetty R., Kundu S., Xia W., Bron M., Schuhmann W., Chirila V., Brandl W., Reinecke T., Muhler M. (2009). PtRu nanoparticles supported on nitrogen-doped multiwalled carbon nanotubes as catalyst for methanol electrooxidation. Electrochim. Acta.

[30] Kundu S., Nagaiah T.C., Xia W., Wang Y., Dommele S.V., Bitter J.H., Santa M., Grundmeier G., Bron M., Schuhmann W. (2009). Electrocatalytic activity and stability of nitrogen-containing carbon nanotubes in the oxygen reduction reaction. J. Phys. Chem. C.

[31] Lei Z., An L., Dang L., Zhao M., Shi J., Bai S., Cao Y. (2009). Highly dispersed platinum supported on nitrogen-containing ordered mesoporous carbon for methanol electrochemical oxidation. Microporous Mesoporous Mater..

[32] Ozaki J.-I., Anahara T., Kimura N., Oya A. (2006). Simultaneous doping of boron and nitrogen into a carbon to enhance its oxygen reduction activity in proton exchange membrane fuel cells. Carbon.

[33] Wu G., Li D., Dai C., Wang D., Li N. (2008). Well-dispersed high-loading Pt nanoparticles supported by shell-core nanostructured carbon for methanol electrooxidation. Langmuir.

[34] Roy S.C., Christensen P.A., Hamnett A., Thomas K.M., Trapp V. (1996). Direct methanol fuel cell cathodes with sulfur and nitrogen-based carbon functionality. J. Electrochem. Soc..

[35] Negro E., Vezzù K., Bertasi F., Schiavuta P., Toniolo L., Polizzi S., Di Noto V. (2014). Interplay between nitrogen concentration, structure, morphology, and electrochemical performance of PdCoNi “core-shell” carbon nitride electrocatalysts for the oxygen reduction reaction. ChemElectroChem.

[36] Di Noto V., Negro E., Polizzi S., Vezzù K., Toniolo L., Cavinato G. (2014). Synthesis, studies and fuel cell performance of “core-shell” electrocatalysts for oxygen reduction reaction based on a PtNix carbon nitride “shell” and a pyrolyzed polyketone nanoball “core”. Int. J. Hydrogen Energy.

[37] Zhou Y., Pasquarelli R., Holme T., Berry J., Ginley D., O’Hayre R. (2009). Improving PEM fuel cell catalyst activity and durability using nitrogen-doped carbon supports: Observations from model Pt/HOPG systems. J. Mater. Chem..

[38] Mansor N., Jorge A.B., Corà F., Gibbs C., Jervis R., McMillan P.F., Wang X., Brett D.J.L. (2014). Graphitic carbon nitride supported catalysts for polymer electrolyte fuel cells. J. Phys. Chem. C.

[39] Mansor N., Jorge A.B., Corà F., Gibbs C., Jervis R., McMillan P.F., Wang X., Brett D.J.L. (2013). Development of graphitic-carbon nitride materials as catalyst supports for polymer electrolyte fuel cells. ECS Trans..

[40] Zheng Y., Liu J., Liang J., Jaroniec M., Qiao S.Z. (2012). Graphitic carbon nitride materials: Controllable synthesis and applications in fuel cells and photocatalysis. Energy Environ. Sci..

[41] Jorge A.B., Dedigama I., Mansor N., Jervis R., Miller T.S., Corà F., Shearing P., Gibbs C., McMillan P.F., Brett D.J.L. (2015). Graphitic carbon nitride materials for energy applications. ECS Trans..

[42] Mansor N., Jia J., Miller T., Suter T., Jorge A.B., Gibbs C., Shearing P.R., McMillan P.M., Mattevi C., Shaffer M. (2016). Graphitic carbon nitride-graphene hybrid nanostructure as a catalyst support for polymer electrolyte membrane fuel cells. ECS Trans..

[43] Mansor M., Miller T.S., Dedigama I., Jorge A.B., Jia J., Brázdová V., Mattevi C., Gibbs C., Hodgson D., Shearing P.R. (2016). Graphitic carbon nitride as a catalyst support in fuel cells and electrolyzers. Electrochim. Acta.

[44] Miller T.S., Jorge A.B., Suter T.M., Sella A., Cora F., McMillan P.F. (2017). Carbon nitrides: Synthesis and characterization of new class of functional materials. Phys. Chem. Chem. Phys..

[45] Wang X., Maeda K., Thomas A., Takanabe K., Xin G., Carlsson J.M., Domen K., Antonietti M. (2009). A metal-free polymeric photocatalyst for hydrogen production from water under visible light. Nat. Mater..

[46] Jorge A.B., Martin D.J., Dhanoa M.T.S., Rahman A.S., Makwana N., Tang J., Sella A., Corà F., Firth S., Darr J.A. (2013). H_2_ and O_2_ evolution from water half-splitting reactions by graphitic carbon nitride materials. J. Phys. Chem. C.

[47] Wang X., Blechert S., Antonietti M. (2012). Polymeric graphitic carbon nitride for heterogeneous photocatalysis. ACS Catal..

[48] Su F., Mathew S.C., Möhlmann L., Antonietti M., Wang X., Blechert S. (2011). Aerobic oxidative coupling of amines by carbon nitride photocatalysis with visible light. Angew. Chem. Int. Ed..

[49] Liu J., Wang H., Antonietti M. (2016). Graphitic carbon nitride “reloaded”: Emerging applications beyond (photo) catalysis. Chem. Soc. Rev..

[50] Ong W.-J., Tan L.L., Ng Y.H., Yong S.T., Chai S.P. (2016). Graphitic carbon nitride (g-C3N4)-based photocatalysts for artificial photosynthesis and environmental remediation: Are we a step closer to achieving sustainability?. Chem. Rev..

[51] Adams R., Shriner R.L. (1923). Platinum oxide as a catalyst in the reduction of organic compounds. III. Preparation and properties of the oxide of platinum obtained by the fusion of chloroplatinic acid with sodium nitrate. J. Am. Chem. Soc..

[52] Keenan C.W., Giesemann B.W., Smith H.A. (1954). Platinum oxide catalysts. J. Am. Chem. Soc..

[53] Bruce W.F. (1936). The preparation of platinum oxide for catalytic hydrogenations. J. Am. Chem. Soc..

[54] Voorhees V., Adams R. (1922). The use of the oxides of platinum for the catalytic reduction of organic compounds. I. J. Am. Chem. Soc..

[55] Felix C., Maiyalagan T., Pasupathi S., Bladergroen B., Linkov V. (2012). Synthesis and optimisation of IrO_2_ electrocatalysts by Adams’ fusion method for solid polymer electrolyte electrolysers. Micro Nanosyst..

